# An analysis of the burden of migraine and tension-type headache across the global, China, the United States, India and Japan

**DOI:** 10.3389/fpain.2025.1539344

**Published:** 2025-02-03

**Authors:** Rongjiang Xu, Ruonan Zhang, Liang Dong, Xiaonuo Xu, Xiaoping Fan, Jiying Zhou

**Affiliations:** ^1^ Department of Neurology, The First Affiliated Hospital of Chongqing Medical University, Chongqing, China; ^2^ Phase I Clinical Research Center, The First Affiliated Hospital of Chongqing Medical University, Chongqing, China

**Keywords:** global burden of disease, migraine, tension-type headache, prevalence, incidence

## Abstract

**Background:**

Recurrent headaches in headache disorders adversely impact quality of life and job. Migraines and tension-type headache TTH) are the most common primary headaches and a prominent cause of disability globally. However, few research compare headache illness burden in China, India, the United States (US), and Japan.

**Methods:**

Global and Chinese, the US, Indian, and Japanese migraine and TTH incidence, prevalence, and disability-adjusted life years were taken from the GBD database for 1990–2021. The data is studied utilizing decomposition analysis, health inequality research, joinpoint regression model, and Bayesian Average Annual Percentage Change (BAPC) model.

**Results:**

The study found that migraine mostly affects women aged 15–49, while TTH are evenly distributed across gender and age. The worldwide average annual percentage change (AAPC) in disease-adjusted life years (DALYs) for migraine and TTH from 1990 to 2021 was 0.0357, a statistically significant trend (*p* < 0.001), as determined using joinpoint analysis. China exhibited the quickest rise in migraine and TTH incidence and prevalence, as well as the age-standardized rate (ASR) of DALYs, of the four nations analyzed. The US had the highest value of these indicators. Forecasting models reveal that without policy action, migraine prevalence will grow but TTH prevalence would stay unchanged. Decomposition research showed that population expansion is the major cause of migraines and TTH, which will be slightly alleviated by population aging. Health disparities across economic growth areas lessened between 1990 and 2021, according to the report.

**Conclusion:**

Globally and in China, migraine and TTH incidence and burden have increased since 1990. Migraines are becoming more common in young and middle-aged women, so headache treatment professionals should invest more in patient education to raise awareness and improve self-management to reduce disease burden and medical costs.

## Introduction

Headache disorders, which encompass a diverse range of neurological afflictions, rank among the most widespread and debilitating conditions, with nearly half of the global adult population being affected (1). Primary headaches mainly include migraines and TTH and these disorders are not only a cause of pain but also a leading cause of disability worldwide (2). Migraine, a common disabling condition that globally affects 15.2% of the population, is the second cause of health loss in terms of years lived with disability and the first among women (3). Meanwhile, TTH is one of the most prevalent headache disorders globally and poses a significant health burden. According to the Global Burden of Disease (GBD) Study 2019, the global prevalence of active headache disorders is 52.0%, with the prevalence of TTH at 26.0% (4). To understand both of these headache disorders, it is important to recognize their epidemiology, and the heavy burden they place on society.

The Global Burden of Disease (GBD) is a systematic scientific endeavor coordinated by the Institute for Health Metrics and Evaluation (IHME) at the University of Washington. Its primary purpose is to quantify the comparative magnitude of health losses due to diseases, injuries, and risk factors across age, gender, and geography at a given point in time (5). The GBD provides a comprehensive set of estimates of all-cause mortality, deaths due to disease, years of life lost (YLLs) due to premature deaths, years lived with disability (YLDs), and disability-adjusted life-years (DALYs) (5). This enables policymakers, health sector leaders, researchers and the public to compare the impact of conditions on health and to understand the most significant factors contributing to health loss in specific locations and populations.

This research evaluated the epidemiology of migraine and TTH in four designated nations: China, India, the United States, and Japan, as well as on a global scale. These four countries were chosen because of their importance and representation in global health research, due to their large population sizes, different levels of economic development, diverse health care systems, cultural and lifestyle differences, and the diversity of etiologic structures. Despite the growing interest in migraine and TTH in global health topics, detailed epidemiologic data on these disorders are still insufficient. By comparing the incidence of migraine and TTH in these four countries, the study not only reveals the epidemiological characteristics and challenges of these two headache disorders in different socioeconomic contexts, but also promotes international collaboration and knowledge sharing in the fields of public health, disease control, and medical research. In addition, the study provides valuable insights for the development of global strategies for the prevention and control of migraine and TTH and the strengthening of public health policies.

## Methods

### Data source and download

We obtained the population, prevalence, incidence and DALYs, and age-standardized rates (ASR) data for all age and age-standardized groups across China, Japan, the US, India and global from GBD 2021 (http://ghdx.healthdata.org/gbd-results-tool, Accessed 31 October 2024). GBD 2021 provides comprehensive data on the burden of 371 diseases and injuries covering 204 regions and countries from 1990 to 2021, as well as data on 88 risk factors. In conducting the study, we strictly adhered to the Strengthening the Reporting of Observational Studies in Epidemiology (STROBE) guidelines.

### Disease definition

In the GBD 2021 cause hierarchy, headache disorders are categorized as level 3, nested under level 2 neurological disorders, with non-communicable diseases at level 1. Under level 4, headache disorders are further differentiated into migraine and TTH, with no subsequent subdivision (6). Migraine is a primary headache disorder usually characterized by recurrent episodes of moderate or severe unilateral throbbing headache. TTH presents as dull, non-throbbing, diffuse or band-like or vice-like pain ranging in intensity from mild to moderate and usually located in the head or neck. In our study, we used codes from the International Classification of Diseases, 10th edition (ICD-10), specifically G43–G43.919, G44.2–G44.229, and G44.4–G44.41, to denote migraine and TTH (7, 8).

### Statistical analysis

#### Joinpoint regression model

To analyze temporal trends in migraine and TTH burden from 1990 to 2021, we used a joinpoint regression model to calculate the annual percentage change (APC) and the average annual percentage change (AAPC) over the entire study period and to determine their corresponding 95% confidence intervals (9). This trend was determined by analyzing the estimated AAPC and its 95% confidence intervals. These trends were then categorized as increasing (AAPC greater than 0), decreasing (AAPC less than 0), or stable (95% confidence intervals of 0) (9). The Joinpoint Regression Program and R software were used to perform data analysis. Statistical significance was defined as a *P* value of less than 0.05.

#### BAPC forecasting model

We adopted the BAPC method for estimating marginal posterior distributions. This method takes advantage of integrated nested Laplace approximations (INLA) and circumvents the typical difficulties encountered with Markov chain Monte Carlo methods in Bayesian analysis (10). The BAPC method has been extensively utilized in analyzing the trends of chronic diseases as well as predicting future disease burden. Therefore, we used the R packages BAPC and INLA in the Integrated Nested Laplace Approximation (INLA) to predict the incidence and burden of DALYs for migraine and TTH in China, the United States, Japan, India, and global for the years 2022–2050. All data analyses were performed using the open source software R (version 4.4.1).

### Decomposition analysis

Decomposition analysis was conducted to determine the effects of population growth, aging, and epidemiological shifts on disease burden (11). Decomposition analysis was conducted to elucidate the major factors contributing to changes in migraine and TTH disease burden between 1990 and 2021. Population expansion pertains to the modifications in the total population size that exert an impact on the disease burden. Specifically, a rapid rise in population can exacerbate the disease burden even when the incidence and mortality rates stay unchanged. Population ageing represents the phenomenon whereby a growing proportion of the elderly within the population is likely to result in a heavier burden of chronic and non-communicable diseases.

Epidemiological shifts denote the alterations in the incidence or mortality rates of diseases, which mirror the advancements in medical technology and public health.

### Health inequality analysis

Health inequality analyses are used to assess changes in health status across populations, aiming to understand the correlations between characteristics such as socioeconomic level, geographic location, gender, and age, and their impact on health outcomes (12). In this study we quantified inequalities in the distribution of the burden of disease for migraine and TTH between countries using an inequality slope index and a concentration index, representing absolute and relative gradient inequalities, respectively (13). These analyses help to identify the drivers of global health inequalities and guide resource allocation to reduce disparities.

In the present study, all counts and rates are reported with 95% uncertainty intervals (UIs), which were generated by employing the 2.5th and 97.5th percentile values obtained from 1,000 ordered draws of the posterior distribution (14). In the Bayesian framework, the Uncertainty Interval, also referred to as the Credible Interval, is constructed under the premise that there is a 95% probability that the true population mean lies within this interval.

## Results

### Burden of migraine and TTH

Statistics collected in 2021 reveal the significant impact of migraine and TTH in different countries, including incidence, prevalence and disability-adjusted life years (DALYs).

#### Migraine

Migraine poses a major challenge to public health systems in several countries around the world, especially China, India and the United States, which are known for their large disease burden. According to 2021 statistics, India ranks first in the world with 8,096,330.9 disability-adjusted life Years (DALYs) (95% UI: 10,157,454.4–18,217,286.6); China followed with 698,198.6 DALYs (95% UI: 11,333,187.7–15,186,289.3); the United States ranked third with 2,129,026.0 DALYs (95% UI: 350,575.2–4,628,603.3); Japan ranked fourth with 545,229.1 DALYs (95% UI: 141,779.6–1,160,180.2).The number and incidence of migraine, corresponding to disability-adjusted life years, also reflect the high health burden of the disease in these four countries. In 2021, the number of people suffering from migraine in India, China, the United States and Japan is 223,124,203.3 (95% UI: 189,835,219.6–254,668,065.2), 184,752,280.1 (95% UI: 160,836,524.7–213,633,958.3), 57,652,573.1 (95% UI: 50,084,784.2–66,079,074.3) and 13,831,413.4 (95% UI: 12,019,193.7–15,876,262.5). In the same year, the new cases of migraine in these four countries were 18,411,214.6 (95% UI: 1,613,821.3–20,773,298.3), 13,047,220.7 (95% UI: 11,597,731.5–14,698,852.1), 3,851,991.4 (95% UI: 3,433,328.5–4,366,538.9) and 840,430.6 (95% UI: 743,167.9–946,923.5). These data highlight the profound impact of migraine on individual health in these countries (Table 1).

**Table 1:** Prevalence, incidence and DALYs of migraine between 1990 and 2021.

Migraine					
	China	US	India	Japan	Global
Incidence					
Number_95% UI_1990	11,518,097.6 (10,091,942.1–13,156,841.9)	3,301,426.0 (2,916,926.6–3,705,168.1)	11,265,219.7 (9,867,461.1–12,808,613.4)	1,022,690.5 (900,054.0–1,161,316.5)	63,496,590.8 (55,194,751.5–72,208,003.4)
ASR	917.3 (808.4–1,037.0)	1,346.6 (1,182.1–1,509.8)	1,231.7 (1,085.8–1,382.5)	851.1 (747.2–964.5)	1,136.9 (995.1–1,287.8)
Number_95% UI_2021	13,047,220.7 (11,597,731.5–14,698,852.1)	3,851,991.4 (3,433,328.5–4,366,538.9)	18,411,214.6 (16,138,211.3–20,773,298.3)	840,430.6 (743,167.9–946,923.5)	90,183,386.9 (78,857,600.5–101,838,162.5)
ASR	975.6 (862.3–1,102.1)	1,322.7 (1,174.2–1,502.6)	1,232.9 (1,080.9–1,385.3)	873.6 (767.2–994.2)	1,153.2 (1,006.1–1,304.5)
AAPC, 1990–2021	0.1976*	−0.0569*	0.0021	0.0841*	0.0455*
*P* value	<0.001	<0.001	0.203282	<0.001	<0.001
Prevalence					
Number_95% UI_1990	133,474,536.5 (114,199,443.7–153,482,597.7)	45,796,590.0 (39,783,190.7–52,334,521.0)	118,839,677.9 (101,287,742.9–137,816,947.4)	14,412,656.6 (12,488,922.9–16,423,840.1)	732,564,462.7 (624,559,243.9–847,058,436.3)
ASR	10,948.5 (9,428.8–12,586.1)	17,098.1 (14,778.2–19,549.9)	14,850.8 (12,801.3–16,953.8)	10,554.9 (9,098.2–12,108.9)	14,027.6 (12,063.4–16,078.1)
Number_95% UI_2021	184,752,280.1 (160,836,524.7–213,633,958.3)	57,652,573.1 (50,084,784.2–66,079,074.3)	223,124,203.3 (189,835,219.6–254,668,065.2)	13,831,413.4 (12,019,193.7–15,876,262.5)	1,158,432,823.8 (995,861,966.4–1,331,312,506.1)
ASR	11,777.5 (10,137.6–13,538.6)	16,750.2 (14,529.8–19,303.4)	14,909.2 (12,771.0–16,967.7)	10,818.4 (9,282.5–12,479.9)	14,246.5 (12,194.1–16,378.7)
AAPC, 1990–2021	0.2356*	−0.0682*	0.0114*	0.0797*	0.0506*
*P* value	<0.001	<0.001	<0.001	<0.001	<0.001
DALYs					
Number_95% UI_1990	5,028,787.5 (767,668.5–11,262,271.4)	1,704,148.2 (245,770.4–3,727,734.5)	4,278,545.3 (497,814.1–9,617,566.4)	562,529.7 (134,458.3–1,204,750.3)	27,412,196.3 (4,076,605.0–60,325,805.8)
ASR	413.0 (66.2–911.0)	635.4 (89.7–1,394.9)	536.0 (69.6–1,181.7)	406.4 (89.5–882.8)	526.8 (83.4–1,145.9)
Number_95% UI_2021	6,988,198.6 (1,133,318.7–15,186,289.3)	2,129,026.0 (350,575.2–4,628,603.3)	8,096,330.9 (1,015,745.4–18,217,286.6)	545,229.1 (141,779.6–1,160,180.2)	43,378,889.8 (6,732,642.2–95,079,454.1)
ASR	443.7 (66.9–971.7)	614.7 (89.7–1,356.0)	541.0 (70.3–1,209.9)	415.3 (88.8–920.2)	532.7 (80.6–1,167.7)
AAPC, 1990–2021	0.2325*	−0.1049*	0.0290*	0.0737*	0.0357*
*P* value	<0.001	<0.001	<0.001	<0.001	<0.001

* and bold indicate that the p-value is less than 0.05.

The ASR analysis further highlights the widespread impact of migraine: the United States, China, India and Japan. In the United States, the ASR for DAYLs was 614.7 (95% UI: 89.7–1,356.0) per 100,000 people, the ASR for prevalence was 16,750.2 (95% UI: 14,529.8–19,303.4), and the incidence was 1,322.7 (95% UI: 1,174.2–1,502.6), the three indicators ranked first in the four countries. In India, the ASR for DAYLs was 541.0 (95% UI: 70.3–1,209.9) per 100,000 population, the ASR for prevalence was 14,909.2 (95% UI: 12,771.0–16,967.7), and the incidence was 1,232.9 (95% UI: 1,080.9–1,385.3). In China, the ASR of DAYLs was 443.7 (95% UI: 66.9–971.7) per 100,000 people, the prevalence of ASR was 11,777.5 (95% UI: 10,137.6–13,538.6), and the incidence was 975.6 (95% UI: 862.3–1,102.1). In Japan, the ASR for DAYLs was 415.3 (95% UI: 88.8–920.2) per 100,000 population, the ASR for prevalence was 10,818.4 (95% UI: 9,282.5–12,479.9), and the incidence was 873.6 (95% UI: 767.2–994.2), the three indicators are the lowest among the four countries. These data provide further evidence of the significant impact of migraine on the health of populations in the United States, China, India, and Japan (Table 1).

#### TTH

TTH, like migraine, has a significant impact on the health of residents of China, India and the United States. According to 2021 statistics, India has the highest number of disability-adjusted life years (DALYs) at 758,396.1 (95% UI: 204,152.4–2,689,271.0). China followed with 716,164.9 DALYs (95% UI: 224,403.1–2,174,717.3), ranking second; the United States ranked third with 261,390.9 DALYs (95% UI: 72,044.4–915,566.6); Japan ranked fourth with 96,075.2 DALYs (95% UI: 28,637.8–300,063.0).The number and incidence of TTH in relation to disability-adjusted life years also reflect the significant health burden of the disease in all four countries. The number of people suffering from TTH in India, China, the US and Japan in 2021 is 373,405,604.7 (95% UI: 329,155,714.7–422,556,131.9), 283,814,151.0 (95% UI: 251,438,661.8–320,431,556.8), 121,925,103.6 (95% UI: 109,446,201.4–135,070,007.6) and 41,142,903.9 (95% UI: 36,598,391.7–45,397,123.3). In the same year, the new cases of TTH in these countries were 137,268,583.8 (95% UI: 119,809,088.5–154,892,262.3), 102,020,341.6 (95% UI: 88,865,128.1–115,000,579.6), 41,482,841.8 (95% UI: 36,422,195.1–46,307,914.2) and 14,077,040.8 (95% UI: 12,378,941.3–15,643,369.9). These data highlight the profound impact of TTH on individual health, particularly in these countries.

The ASR analysis further highlights the broad impact of TTH in four countries: the United States, China, India, and Japan. In the United States, the ASR for DAYLs was 70.3 (95% UI: 18.1–258.3) per 100,000 people, the ASR for prevalence was 34,320.6 (95% UI: 30,762.9–38,348.9), and the incidence was 12,110.7 (95% UI: 10,550.6–13,520.5), the three indicators ranked first in the four countries. In India, the ASR for DAYLs was 51.9 per 100,000 population (95% UI: 14.1–180.6), the prevalence was 25,298.0 (95% UI: 22,421.8–28,418.0), and the incidence was 9,333.4 (95% UI: 14.1–180.6) 8,160.5–10,426.4). In China, the ASR of DAYLs per 100,000 population was 43.5 (95% UI: 13.1–141.3), the prevalence of ASR was 18,525.1 (95% UI: 16,380.9–20,958.7), and the incidence was 6,851.1 (95% UI: 5,957.5–7,729.6). In Japan, the ASR for DAYLs was 64.6 per 100,000 population (95% UI: 17.9–215.3), the prevalence was 29,705.7 (95% UI: 26,600.4–33,406.2) and the incidence was 10,726.1 (95% UI: 17.9–215.3) 9,392.8–12,010.9). These data provide further evidence of the significant impact of TTH on the health of populations in the United States, China, India, and Japan.

Together, these data illustrate the great impact of migraine and TTH on people's health in the United States, China, India and Japan. While age-standardised rates vary in other countries around the world, the significant impact of migraine and TTH on health in these four countries remains profound. This highlights the urgent need to implement effective migraine and TTH prevention and treatment measures globally, with a particular focus on countries with the highest disease burden.

### Changes in the burden of migraine and TTH 1990–2021

**Figure 1:**
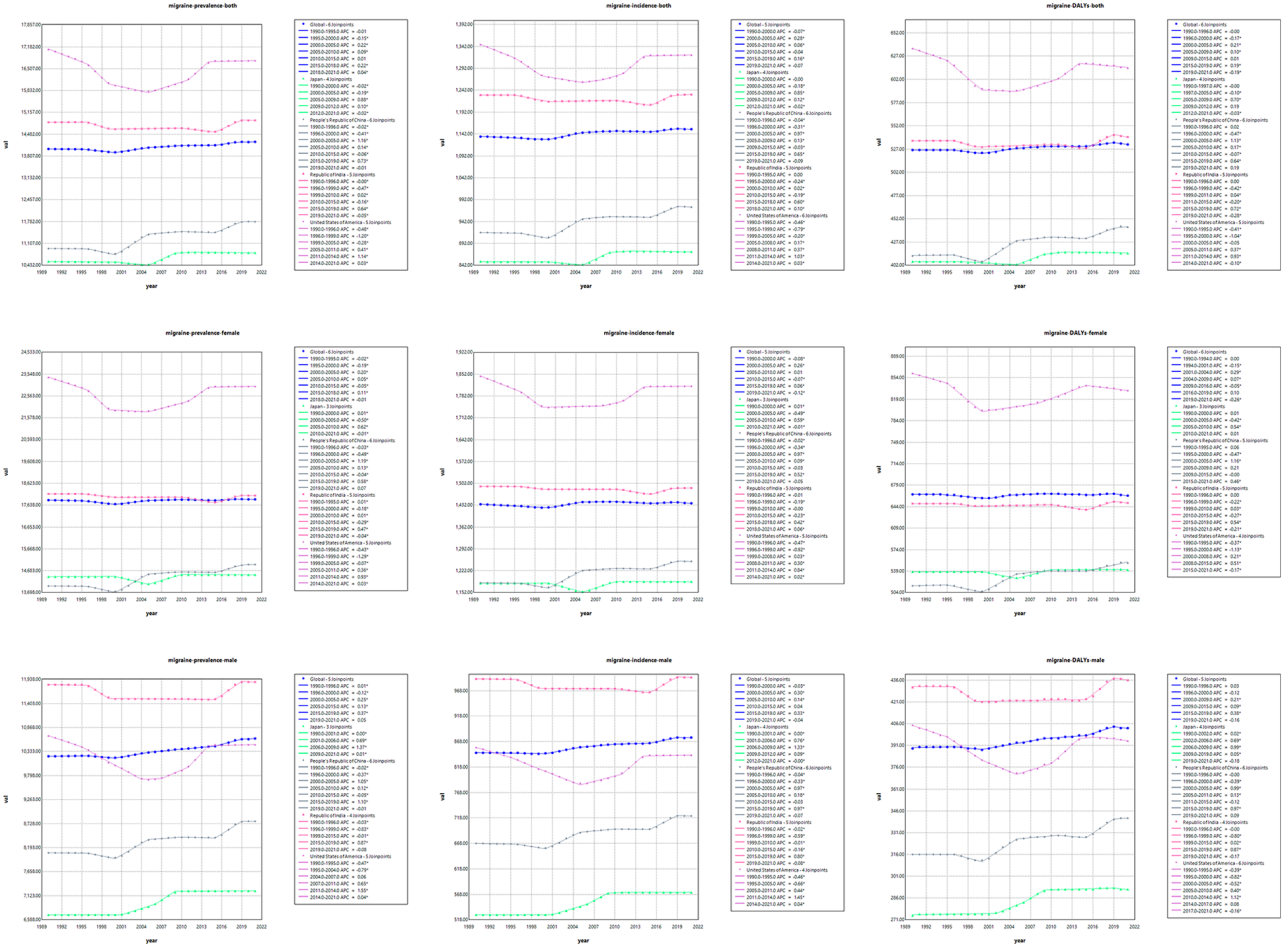
Trends in ASIR, ASPR and age-standardized DALYs rate for migraine.

AAPC results showed that the global burden of disease caused by migraine increased between 1990 and 2021, with a global AAPC of 0.0357 for the ASR of DALYs (*P* < 0.001). Among the four countries, China showed the fastest increase in disease burden, with an AAPC of 0.2325 for DALY (*P* < 0.001). The AAPC for the ASR of DALYs in Japan and India is 0.0290 (*P* < 0.001) and 0.0737 (*P* < 0.001), respectively, with a slow growth rate. The ASR for DALY in the United States was −0.1049 (*P* < 0.001), with a slow decline. However, due to the growth of the absolute number of population, the absolute value of DALY has increased correspondingly. Similarly, there are similar trends in the incidence and prevalence of migraine. From 1990 to 2021, the joinpoint regression model plot of each burden indicator for migraine contains the following turning points (Table 1; Figure 1).

**Figure 2:**
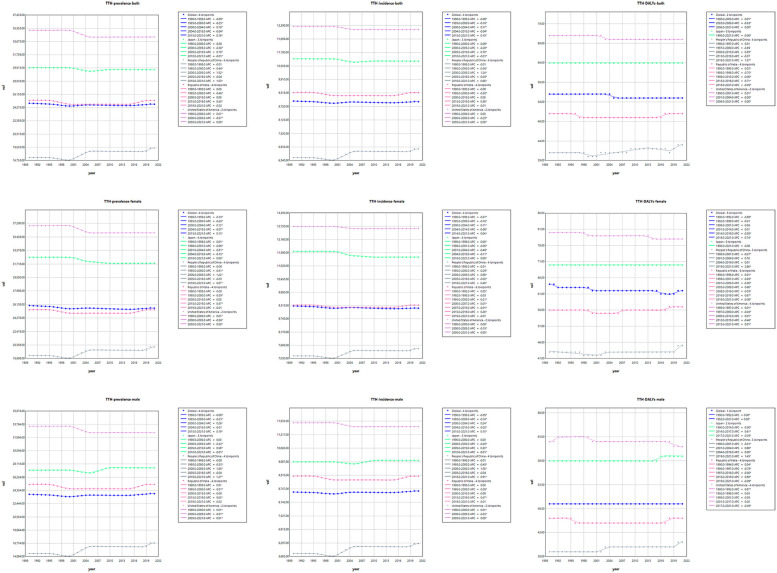
Trends in ASIR, ASPR and age-standardized DALYs rate for TTH.

Over the period 1990–2021, the global burden of disease caused by TTH showed a slight downward trend, in contrast to the changing pattern of migraine. Annual percentage change in age-standardised rates show that the global AAPC for disability-adjusted life years (DALYs) due to TTH is −0.0733 (*P* < 0.001), indicating a reduction in disease burden. Among the four countries, the TTH disease burden increased fastest in China, with AAPC of 0.1515 for DALY (*P* < 0.001); in India, the growth rate is slower, the AAPC for ASR for DALY is 0.0421 (*P* < 0.001); the ASR for DALY in the United States was −0.0643 (*P* < 0.001), showing a slow downward trend. However, due to the growth of the absolute number of population, the absolute value of DALY increases correspondingly. Similarly, the incidence and prevalence of migraine have shown a similar trend. From 1990 to 2021, the joinpoint regression model plot of each disease burden indicator for TTH can be seen in Table 2; Figure 2.

**Table 2:** Prevalence, incidence and DALYs of TTH between 1990 and 2021.

TTH					
	China	US	India	Japan	Global
Incidence					
Number_95% UI_1990	76,930,406.5 (66,380,713.5–87,690,508.6)	32,100,028.7 (27,902,975.9–36,149,161.9)	76,633,070.8 (66,322,990.8–86,338,287.5)	14,356,011.6 (12,665,596.6–15,913,819.5)	470,298,233.1 (408,471,892.0–527,847,536.3)
ASR	6,467.4 (5,603.1–7,291.7)	12,248.2 (10,580.9–13,784.7)	9,335.1 (8,162.3–10,430.6)	10,832.4 (9,538.9–12,085.5)	8,960.3 (7,815.1–10,074.3)
Number_95% UI_2021	102,020,341.6 (88,865,128.1–115,000,579.6)	41,482,841.8 (36,422,195.1–46,307,914.2)	137,268,583.8 (119,809,088.5–154,892,262.3)	14,077,040.8 (12,378,941.3–15,643,369.9)	719,043,093.3 (629,219,079.9–804,949,048.8)
ASR	6,851.1 (5,957.5–7,729.6)	12,110.7 (10,550.6–13,520.5)	9,333.4 (8,160.5–10,426.4)	10,726.1 (9,392.8–12,010.9)	8,931.3 (7,788.2–10,020.8)
AAPC, 1990–2021	0.1902*	−0.0359*	−0.0025	−0.0316*	−0.0074
*P* value	<0.001	<0.001	0.596978	<0.001	0.105206
Prevalence					
Number_95% UI_1990	204,064,313.2 (176,898,604.5–233,568,232.2)	94,542,714.6 (84,760,567.2–105,559,759.2)	200,920,225.7 (175,295,998.0–229,590,953.1)	40,991,889.7 (36,541,697.0–45,620,021.7)	1,286,366,671.7 (1,122,503,420.8–1,467,160,187.5)
ASR	17,174.5 (15,086.7–19,379.7)	35,290.0 (31,611.5–39,433.1)	25,300.0 (22,420.1–28,440.7)	29,994.3 (26,619.9–33,614.9)	24,904.8 (21,960.0–28,038.8)
Number_95% UI_2021	283,814,151.0 (251,438,661.8–320,431,556.8)	121,925,103.6 (109,446,201.4–135,070,007.6)	373,405,604.7 (329,155,714.7–422,556,131.9)	41,142,903.9 (36,598,391.7–45,397,123.3)	2,011,612,877.5 (1,776,544,390.8–2,270,860,638.8)
ASR	18,525.1 (16,380.9–20,958.7)	34,320.6 (30,762.9–38,348.9)	25,298.0 (22,421.8–28,418.0)	29,705.7 (26,600.4–33,406.2)	24,764.8 (21,863.6–27,954.7)
AAPC, 1990–2021	0.2516*	−0.0884*	−0.0028	−0.0311*	−0.0141*
*P* value	<0.001	<0.001	0.654049	<0.001	0.031872
DALYs					
Number_95% UI_1990	489,462.7 (151,736.8–1,687,075.4)	195,297.0 (50,503.7–716,795.1)	384,655.5 (99,773.1–1,487,621.4)	92,878.1 (26,530.0–295,778.1)	2,848,687.6 (820,890.8–9,563,745.4)
ASR	41.8 (13.1–141.4)	71.7 (18.1–266.5)	51.2 (14.0–187.4)	64.6 (17.9–216.5)	57.0 (16.8–186.1)
Number_95% UI_2021	716,164.9 (224,403.1–2,174,717.3)	261,390.9 (72,044.4–915,566.6)	758,396.1 (204,152.4–2,689,271.0)	96,075.2 (28,637.8–300,063.0)	4,596,785.3 (1,347,300.8–15,012,932.7)
ASR	43.5 (13.1–141.3)	70.3 (18.1–258.3)	51.9 (14.1–180.6)	64.6 (17.9–215.3)	55.7 (16.1–185.1)
AAPC, 1990–2021	0.1515*	−0.0643*	0.0421*	−0.001	−0.0733*
*P* value	<0.001	<0.001	<0.001	0.73102	<0.001

### Distribution of migraine and TTH burden across different age groups in 2021

**Figure 3:**
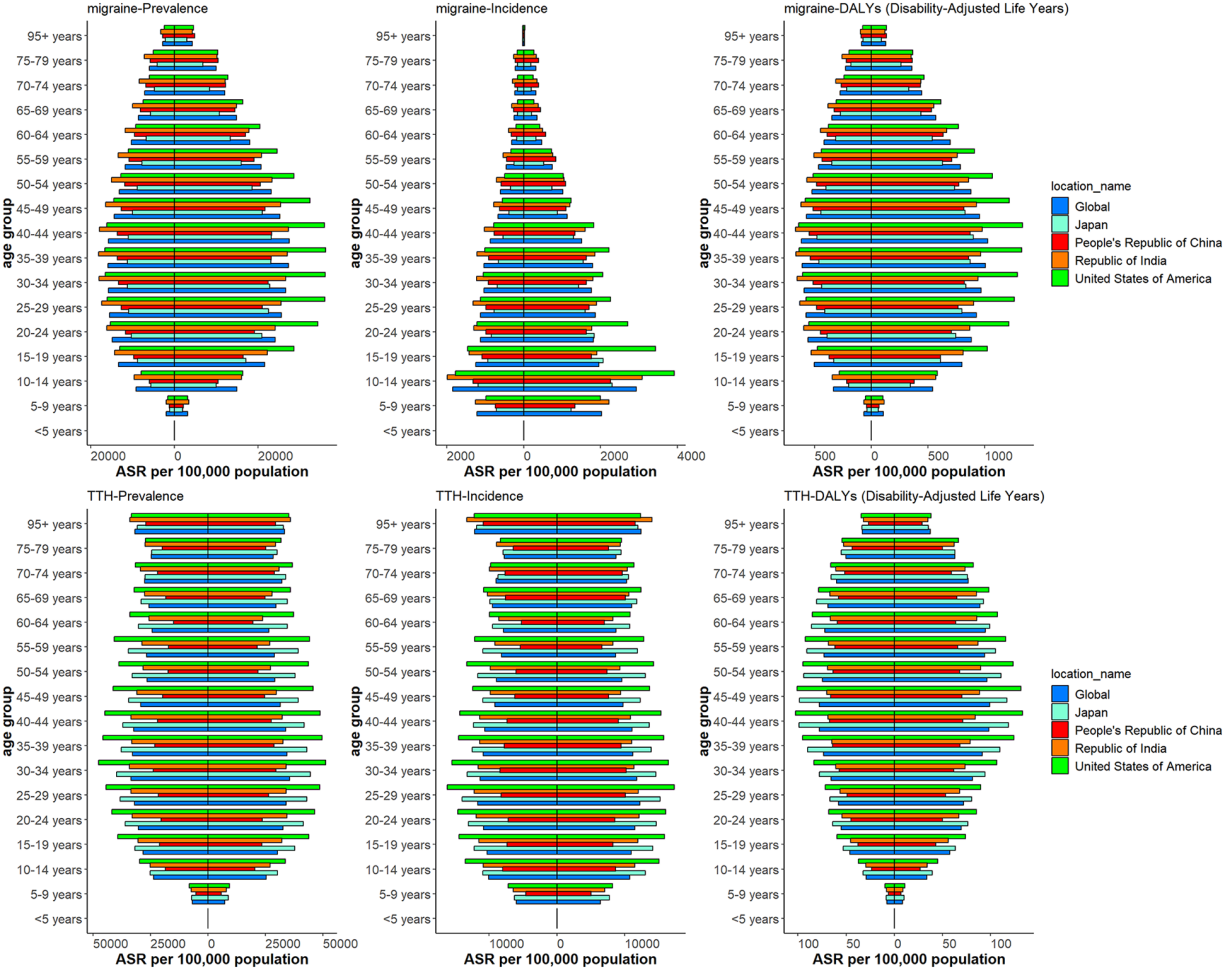
Distribution of migraine and TTH prevalence, incidence and DALYs by age and gender. Female: Right of the 0 scale; Male: Left of the 0 scale.

The study provides a comprehensive analysis of the disease burden of migraine and TTH across age groups and genders in 2021 globally and in four specific regions in China, the United States, India and Japan. The assessment measures included disability-adjusted life years (DALYs), prevalence and incidence. The results showed that between 1990 and 2021, the global incidence of migraine DALYs was higher in women than in men, peaking in the 40–44 age group and subsequently decreasing with age. The onset of migraine in female peaked at 10–14 years of age, and then slowly decreased. The age of migraine in female is mainly between 15 and 49 years old. In contrast, the three indicators of migraine in male are lower than in female, and the middle-aged and young people are mainly, but the distribution is relatively uniform across all ages. The age and gender distribution of TTH is different from that of migraine, and the disease burden of female is slightly higher than male, and affects all ages (Figure 3).

### Migraine and TTH development forecast

**Figure 4:**
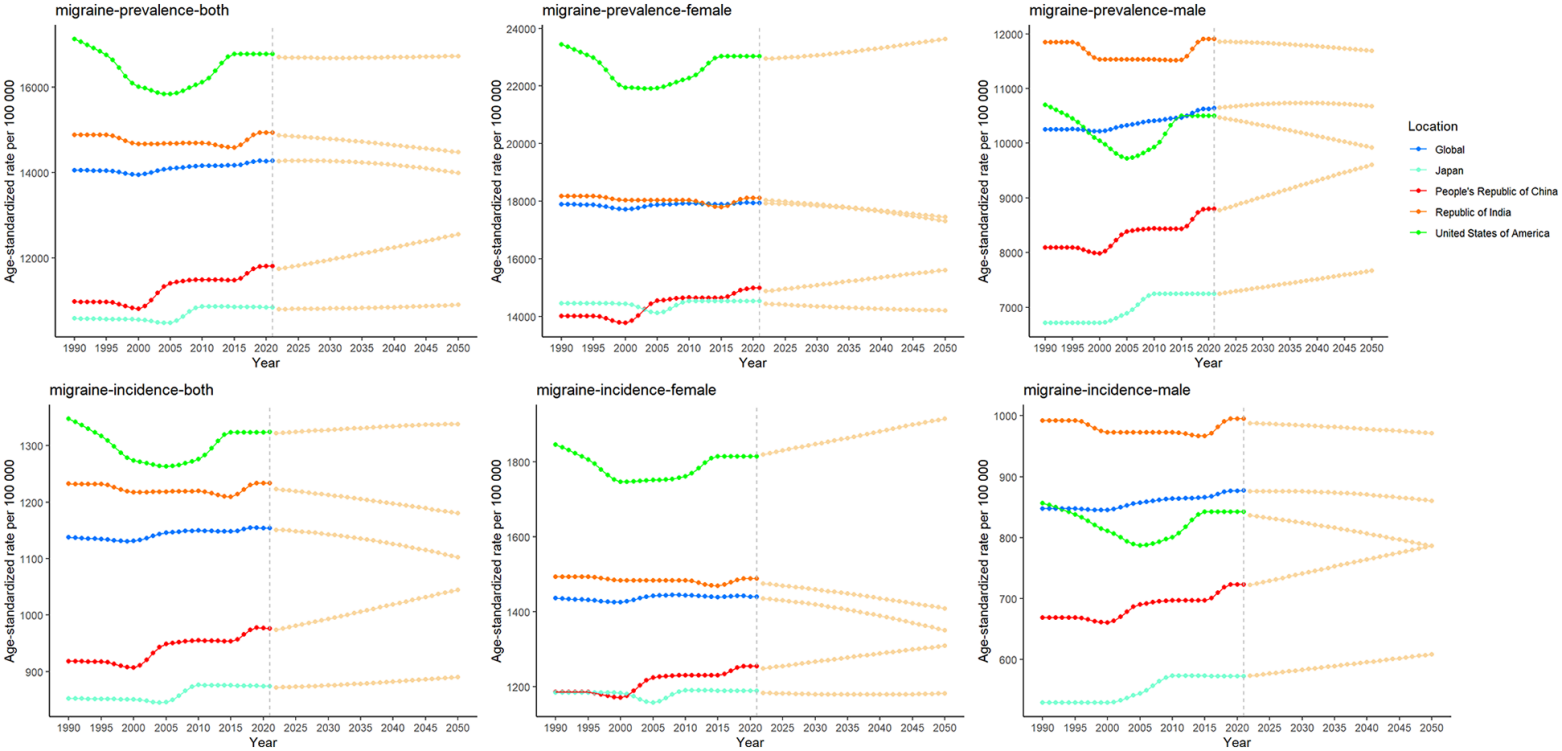
Future forecasts of the prevalence and incidence of migraine.

**Figure 5:**
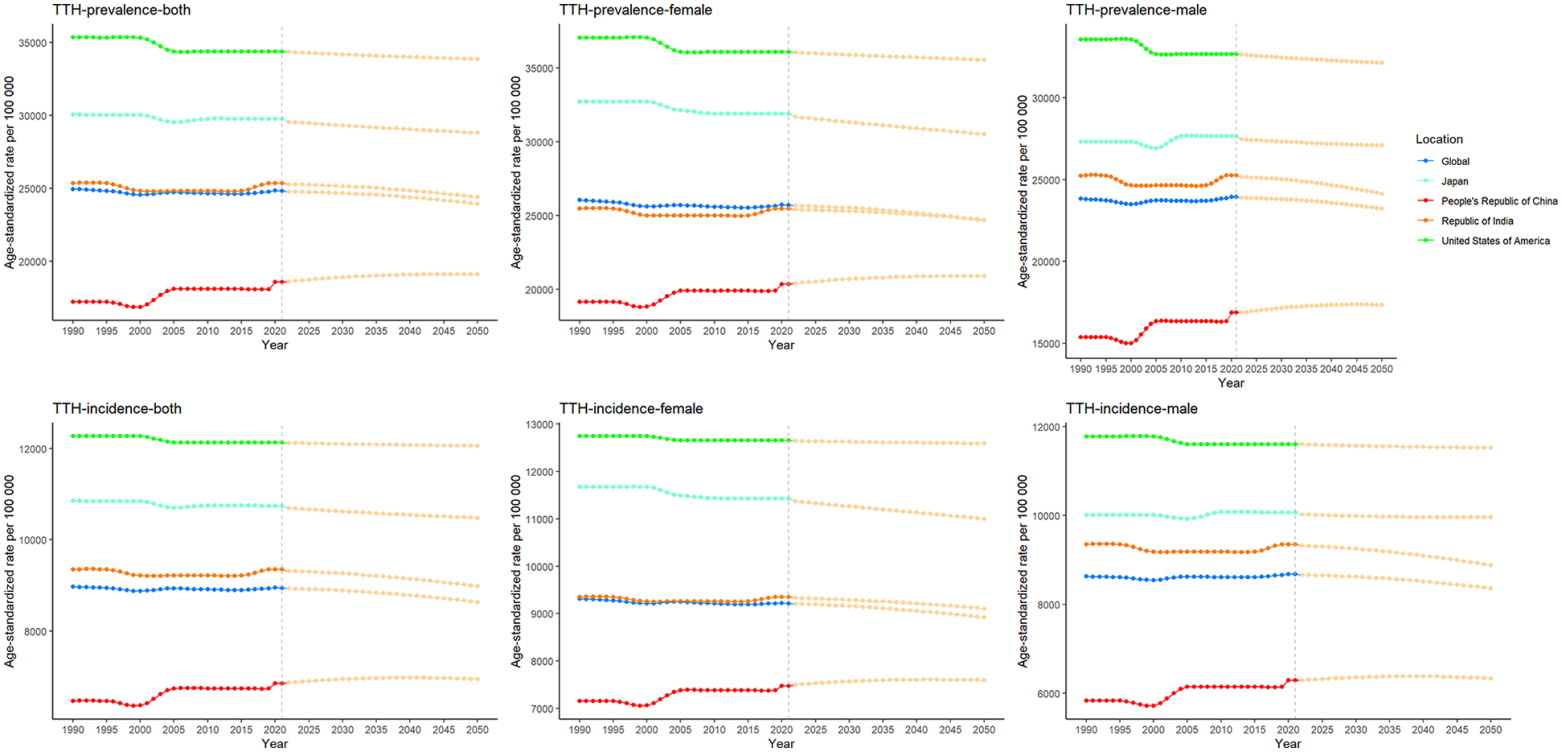
Future forecasts of the prevalence and incidence of TTH.

In Figure 4, we show the evolution of projected prevalence and incidence of migraine in China, the United States, India, Japan, and globally, both in men and women. The study showed that the age-standardized incidence (ASIR) and age-standardized prevalence (ASPR) of migraine in China showed a marked upward trend, and the trend was similar in male and female groups. In the United States, ASIR and ASPR are increasing in both women and total population, while decreasing in men. ASIR and ASPR of total population and male have increased in Japan, in contrast, the female population has shown a slow decline. The trends were similar globally and in India, with ASIR and ASPR decreasing in both women and total population, and slightly increasing in men. In Figure 5, we present a trend analysis of TTH between 2022 and 2050. The results show that in the five countries, the changes in both incidence and prevalence were small, and the data between the sexes showed only a slight upward or downward trend.

### Decomposition analysis of migraine and TTH

**Figure 6:**
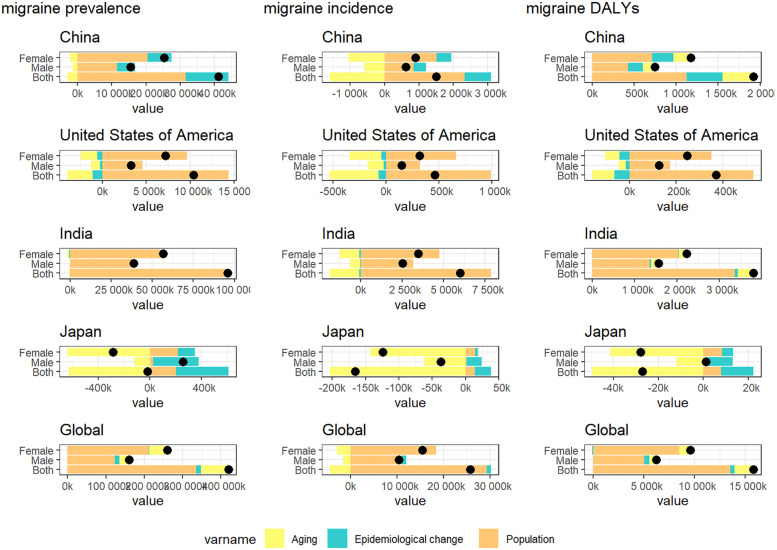
Changes in migraine prevalence, incidence and DAYLs from 1990 to 2021 are influenced by population growth, aging, and epidemiologic changes. Black dots indicate the cumulative effect of all three determinants. For each determinant, a positive value indicates that the factor led to an increase in incidence and a negative value indicates that the determinant led to a decrease in incidence.

**Figure 7:**
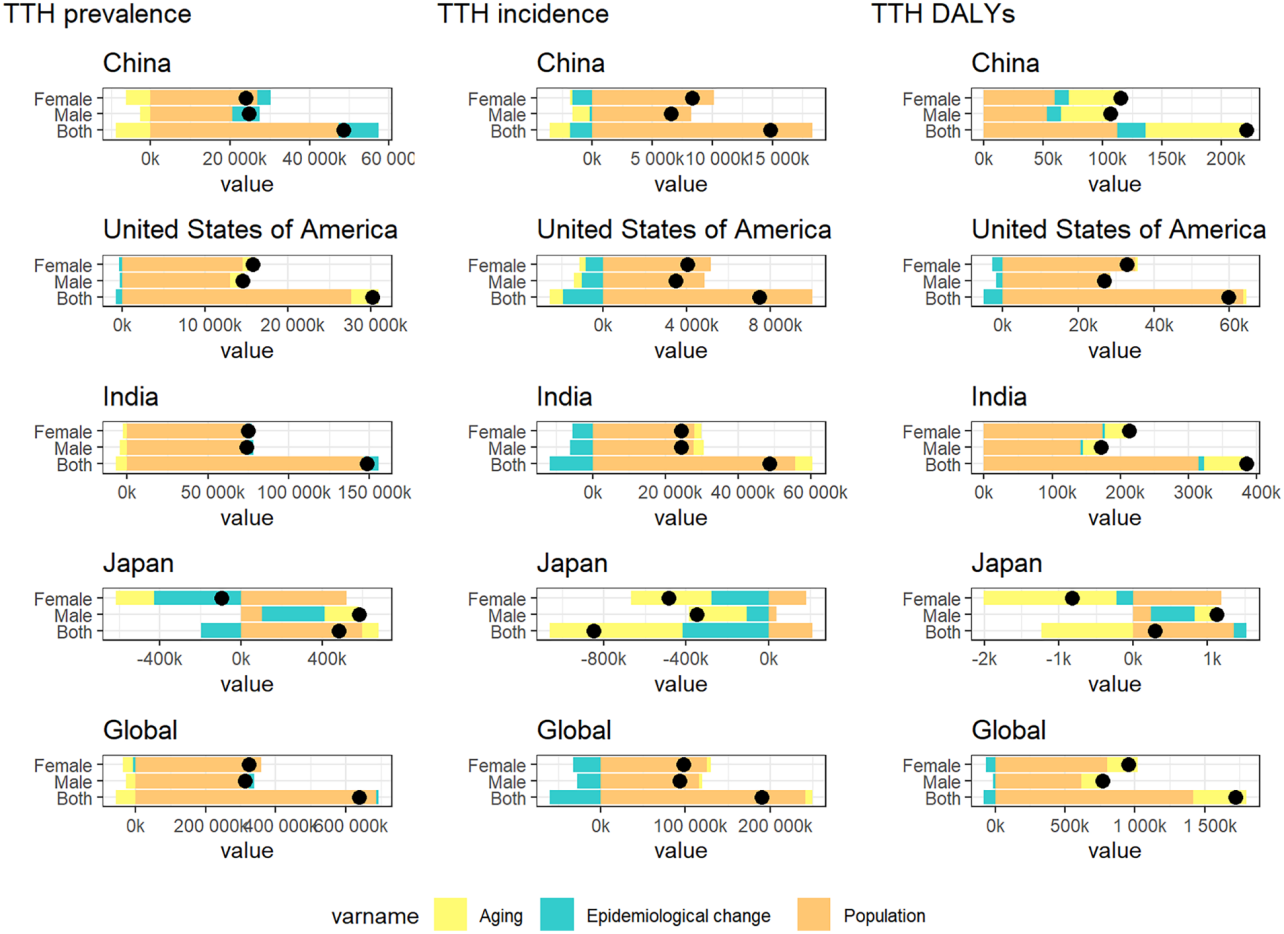
Changes in TTH prevalence, incidence and DAYLs from 1990 to 2021 are influenced by population growth, aging, and epidemiologic changes. Black dots indicate the cumulative effect of all three determinants. For each determinant, a positive value indicates that the factor led to an increase in incidence and a negative value indicates that the determinant led to a decrease in incidence.

In order to further explore the relative impact of population expansion, aging process, and shifting epidemiological patterns on changes in the epidemiological characteristics of migraine andTTH, we conducted a comprehensive disaggregated analysis (15). In China, the United States, and India, the increase in prevalence, incidence and DAYLs is primarily driven by population growth. In Japan, however, the burden of headaches is growing at a relatively slow pace due to the protective effect of an aging population. It is worth noting that India has a lower degree of population aging compared to China and the United States, combined with its rapid population growth, resulting in a migraine burden mainly attributable to population growth (Figure 6). In the decomposition analysis of TTH, we observed a similar trend (Figure 7).

### Health inequality analysis of migraine and TTH

**Figure 8:**
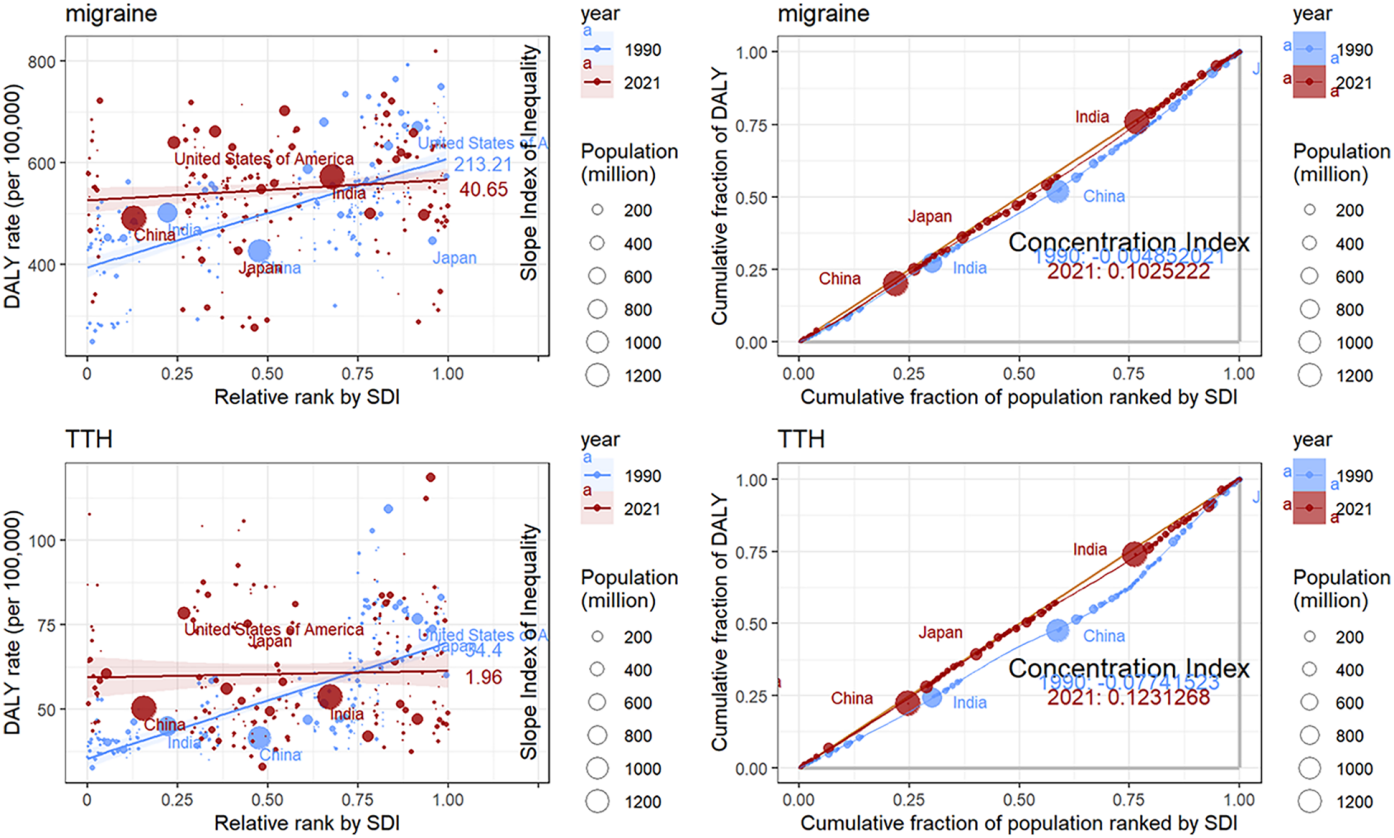
Visualization of the slope index of inequality and concentration index results for DALYs.

Between 1990 and 2021, the global gap in disability-adjusted life years (DALYs) due to migraine and tension TTH has gradually narrowed, but health inequalities due to different levels of economic development remain. For migraine, the slope index of inequality (SII), a measure of health inequality, declined from 213.21 DALYs per 100,000 people in 1990 to 40.65 DALYs per 100,000 people in 2021, indicating an improvement in global health inequality. The Concentration Index (CI), which assesses the concentration of health inequalities, rose from −0.005 in 1990 to 0.10 in 2021. TTH showed a similar pattern to migraine. For TTH, SII declined from 34.40 DALYs per 100,000 people in 1990 to 1.96 DALYs per 100,000 people in 2021, indicating an improvement in global health inequality. The Concentration Index (CI) rose from −0.08 in 1990 to 0.12 in 2021. TTH showed a similar pattern to migraine (Figure 8).

## Discussion

As two common headache types, migraine and TTH pose a heavy disease burden to individuals and society (1, 16). Studies have shown that people with migraine face a higher risk of disability and a lower quality of life than people without headache symptoms (17). Similarly, TTHs are extremely common in adolescents and adults, and have a significant impact on the patient's daily life and ability to work (18). Therefore, effective treatment and management strategies are urgently needed for both headache types.

Using incidence, prevalence, and disability-adjusted life years (DALYs) from global burden of disease (GBD) data over the past 30 years, this study presents a comprehensive picture of trends in the burden of migraine and TTH by year and age, and compares differences between China, the United States, India, and Japan from the GBD database. In terms of migraine, we found that the age-standardized incidence rate (ASIR), age-standardized prevalence rate (ASPR), and ASR for DALYs continue to increase in China until 2050. In contrast, the United States has the highest ASIR, ASPR, and ASR for DALYs among the four countries. In our analysis of TTH, we found that ASIR, ASPR, and ASR for DALYs also continued to increase in China, but none of the four countries showed a significant increase in subsequent projections. As developing countries with the largest and second largest populations respectively, India and China will continue to face a significant burden of migraines and TTHs, and policies will be needed to alleviate this dire situation.

In China, ASPR and ASIR of migraine and TTH have shown a continuous increasing trend, which is in stark contrast to the situation in the other four countries. A combination of population growth and epidemiological changes is driving this growth, although there is some mitigation from population aging. The increase in headache burden is closely related to China's rapid economic development over the past 30 years, which has led to significant changes in demographic and epidemiological characteristics (6). Changes in lifestyle and work habits, such as increased sedentary habits, increased stress, the popularity of high-fat, high-protein diets, and increased use of electronic devices, can trigger and exacerbate headaches (19–21). At the same time, the increase of headache disease burden is also related to the imbalance of medical resources distribution and the gap between urban and rural areas (22). The uneven distribution of medical resources in different regions has led to the inability of headache patients in many rural areas to receive timely and effective treatment, which has led to the development of chronic headache, further aggravating the disease burden of headache.

India also suffers from a huge headache disease burden. The prevalence of migraine and TTH is much higher in India than in China. However, over the past 30 years, the ASPR of migraine and TTH has not increased significantly; According to our BACP forecasting model, the ASPR of migraine and TTH in India is expected to show a downward trend from 2022 to 2050. Therefore, the increase in headache disease burden in India is mainly due to population growth that is not effectively controlled. According to World Population Prospects 2024, India's population growth since 1950 and predicted that India's population would reach 1.7 billion by 2060 (23).

The United States has the highest ASPR of migraine and TTH, with 16,750.2 (range 14,529.8–19,303.4) and 35,290.0 (range 31,611.5–39,433.1) cases per 100,000 people, respectively. This may be due to better medical resources and diagnostic capacity in areas with a high socio-economic development index (SDI), resulting in a higher number of reported cases; At the same time, high-stress work environments and unhealthy lifestyles may also contribute to the high incidence of migraines (10). Recently, the rising trend of prevalence and incidence has gradually leveled off, or even slowly decreased, which may mean that the disease burden has reached a saturation state; as the absolute number of people grows, so does the absolute amount of disease burden. In terms of disability, the United States showed the highest disability-adjusted life years (DALYs) for migraine and TTH among the four countries, which is consistent with the substantial evidence of migraine and TTH related disability in the United States. Unlike China and India, economic and social development in the United States has become more stable, and people's lifestyles and work habits have changed little, which may explain the stable prevalence.

Japan has the lowest migraine prevalence among the four countries, which is inseparable from the country's severe aging population. Japan has the highest proportion of older adults in the world, with 29.8% of the population aged 65 or over in 2021 (24). By 2050, it is projected that 37.5% of the population will be 65 or older (24). In the meantime, Japan's total fertility rate has been consistently low, much below the replacement level for a population, contributing to a rapidly aging and declining population (24). However, unlike migraines, the prevalence of TTH in Japan is second only to the United States, which is mainly influenced by a number of factors. First of all, as a developed country in Asia, Japan has a high level of economic development and a large urban population, and a long time of sitting in urban life and high work pressure are the preconditions for TTH (25). Second, the prevalence of TTH is similar across age groups, so even an aging population has not led to a reduction in headache incidence (26).

This study analyzed ASPR, ASIR, and DAYL associated with migraine and TTH by sex and age globally and in China, the United States, India, and Japan in 2021. We found that women have a significantly higher disease burden than men when it comes to migraines. In terms of age groups, the burden of disease peaked between the ages of 15 and 49 years and then gradually decreased in both directions, a trend consistent with the epidemiological characteristics of migraine (27, 28). In the case of TTH, there was little difference in disease burden between women and men, and the distribution was more uniform across all age groups (29). Because TTHs are characterized by recurrent, relatively non-disabling headaches with fewer accompanying symptoms (30). So it's much less disabling than the migraine.

The health inequality analysis conducted in this study, combined with an examination of migraine and TTH burdens globally as well as in China, the United States, India, and Japan, revealed significant trends in DALY in regions with different socio-demographic characteristics. The slope index results show that the DALY gap between regions with high and low SDI levels is narrowing from 1990 to 2021. The Concentration Index (CI) values changed from negative to positive, suggesting that the burden of migraine disease in 1990 and 2021 was reversed and is now more concentrated in wealthier groups. These findings highlight the complex interplay of socioeconomic factors and the healthcare system in shaping migraine and TTH outcomes.

### Limitations

There are some limitations to the study. First, although the GBD study used as many data sources as possible, data remains scarce in some countries, which can affect the accuracy of disease burden estimates. Second, temporal trends in migraine and TTH may be influenced by changes in the diagnostic level of clinical headache physicians over time. Third, this study relies entirely on the GBD database. Fourth, migraine can be divided into migraine with aura and migraine without aura, episodic migraine and chronic migraine. However, the GBD data only contained data on migraines, not on the various migraine subtypes. Finally, because the GBD database lacks relevant data, it is not possible to study the impact of multiple risk factors on migraine and tension type.

## Conclusions

From 1990 to 2021, the global burden of migraine and TTH is on the rise, with China leading the way. In all age groups, the prevalence of migraine is high in people aged 15–49 years, and the proportion of women is higher than that of men in all age groups. Factors such as rapid urbanization, economic development and high-pressure lifestyle may lead to increased headache burden in some areas, but with the advancement of population aging, the increase of migraine burden gradually leveled off, and had little impact on TTH. Therefore, it is necessary to develop targeted headache prevention strategies, especially in areas where the headache burden continues to increase.

## Data Availability

Publicly available datasets were analyzed in this study. This data can be found here: http://ghdx.healthdata.org/gbd-results-tool.
